# Hospital Rating Organizations’ Quality and Patient Safety Scores: Analysis of Result Discrepancies

**DOI:** 10.1007/s11606-024-08950-0

**Published:** 2024-07-19

**Authors:** Samer Badr, Tareq Nahle, Shakibur Rahman, Amine Al Soueidy, Martha Stefaniak, Marisha Burden, Jean-Sebastien Rachoin

**Affiliations:** 1 Center of Hospital-Based Services, Cooper University Healthcare, Camden, NJ USA; 2 Cooper Medical School of Rowan University, Camden, NJ USA; 3 Universite Saint-Joseph, Beirut, Lebanon; 4 Department of Medicine, Cooper University Healthcare, Camden, NJ USA; 5 Clinical Effectiveness Department, Cooper University Healthcare, Camden, NJ USA; 6 Division of Hospital Medicine, University of Colorado School of Medicine, Aurora, CO USA

**Keywords:** hospital ratings, leapfrog, hospital compare, healthgrades, US news

## Abstract

**Background:**

In the USA, multiple organizations rate hospitals based on quality and patient safety data, but few studies have analyzed and compared the rating results.

**Objective:**

Compare the results of different US hospital-rating organizations.

**Design:**

Observational data analysis of US acute care hospital ratings.

**Participants:**

Four rating organizations: Hospital Compare® (HC), Healthgrades® (HG), The Leapfrog Group® (Leapfrog), and US News and World Report® (USN).

**Main Measures:**

We analyzed the level of concordance (similar ranking), discordance (difference of 1 or more rankings), and severe discordance (difference of two or more rankings), as well as differences and correlations between the scores.

**Key Results:**

From Feb 1 to Oct 3, 2023, we analyzed data from 2,384 hospitals. In Leapfrog, there were 688 hospitals (29%) with Grade A, 652 (27.3%) with B, 885 (37.1%) with C, 153 (6.4%) with D, and 6 (0.3%) with F. For HC, 333 hospitals (14%) had five stars, 676 (28.4%) four, 695 (29.2%) three, 502 (21.4%) two, and 171 (7.2%) one-star. In ratings between HC and Leapfrog, discordance was 70%, and severe discordance was 25.1%. USN ranked 469 hospitals (19.7%). Within the USN-ranked hospital group, there was a 62% discordance and 19.8% severe discordance between HC and Leapfrog. The analysis of orthopedic procedures from HG and USN showed discordance ranging from 48 to 61.2%.

**Conclusion:**

The rating organizations’ reported metrics were highly discordant. A hospital's ranking by one organization frequently did not correspond to a similar ranking by another. The methodology and included timeline and patient population can help explain the differences. However, the discordant ratings may confuse patients and customers.

## INTRODUCTION

The increased emphasis on transparent, publicly accessible data in the USA for the last two decades has allowed patients and customers to compare hospitals and clinicians’ performances across institutions and conditions.^1^ Transparency of healthcare quality is crucial and quality information necessary for patients to choose between providers, insurers to procure care, and providers to improve their services.^2^ Several entities rate hospitals and clinicians on quality and patient safety and update their findings and assessments once or twice yearly^3^.

However, concerns have been raised that a high rating alone is not always associated with better clinical outcomes.^4,5^ The lack of transparent methodology has also created a credibility gap.^6,7^ Hospital ratings by one entity may not necessarily translate into a comparable rating by another, resulting in patient and stakeholder confusion.^3,8,9^

Studies comparing rating systems either included a hospital sample that was too small, focused mainly on highly ranked hospitals,^3,8,9^ or failed to compare the specific components of the scores. Thus, there is a need to perform more studies that include a larger number of hospitals, not only the top-ranked ones, and compare the performance of the ranking systems.

In this study, we aimed to analyze and compare the overall rankings and diagnosis, condition, and procedure-specific scores a hospital receives from four national rating organizations: Hospital Compare® (HC), Healthgrades® (HG), The Leapfrog Group® (Leapfrog), and US News and World Report® (USN).

## METHODS

### Study Design

We performed an observational study and gathered data from four sources: HC, HG, Leapfrog, and USN. We chose those organizations because they collect data on hospitals nationwide, and their findings and ratings are available without specific subscriptions.

The Cooper University Healthcare Institutional Review Board reviewed this study and deemed it exempt from institutional review. We followed the STROBE reporting guidelines for observational studies.^10^

### Data Sources/Databases Searched

HC is a public reporting tool from the Centers for Medicare and Medicaid Services (CMS).^11^ It gathers data from hospitals participating in the Medicare program.

HG is a private US company that evaluates hospitals based on risk-adjusted mortality and in-hospital complications.^12^ It converts data from publicly available sources into a number of stars (maximum 5) for different metrics.

Leapfrog ^13^ is a nonprofit organization that has conducted a national hospital survey twice yearly since 2001. Hospital administrators fill out the surveys, but Leapfrog verifies the accuracy of the information. Leapfrog assesses hospitals in many domains and assigns an overall safety grade from A (highest) to F (Lowest).

USN is a digital media company that publishes rankings on various domains such as education, cars, and health.^14^ It evaluates hospitals on multiple metrics and ranks them regionally and nationally.

### Search Strategy

From Feb 1, 2023, to Oct 3, 2023, we queried quality scores and patient safety data for all the acute care hospitals in the USA. We obtained the list of all the acute care hospitals from the American Hospital Directory, which uses publicly available sources.^15^

We excluded specialty, pediatric, and critical access hospitals and those that did not have at least one data entry in each searched database. We used the physical address to validate hospitals with different names in different databases. In the final sample, we included hospitals that declined to respond to the *Leapfrog hospital survey*® but still received a safety grade. Four authors (AE, JR, TN, and SI) conducted the search, and for each hospital, one author reviewed all four databases simultaneously.

### Variables Recorded

From the Leapfrog database, we recorded the overall hospital safety grade, and in the category “problems with surgery,” the variable “dangerous object left in patient's body’’, the object being a sponge or a tool. We dichotomized these surgical events and analyzed a simplified binary metric where we reclassified any score greater than 0 as Yes or occurred and a score of 0 as No or absent. From HC, we recorded the overall number of stars. From USN and HG, we recorded the score for 30-day mortality for the following conditions: heart attack, aortic or valve surgery, bypass surgery, heart failure, colon or colon cancer surgery, stroke, COPD, and pneumonia. We also recorded each institution's overall score for the following procedures: hip fracture treatment, hip replacement surgery, and knee replacement surgery. HG also reported on surgical objects left in a patient’s body. Finally, from USN, we recorded whether an institution was regionally ranked and was high-performing in a specialty, condition, or procedure.

### Statistical Analysis

We presented categorical variables as numbers (percentages) and continuous variables as mean (± standard deviations). We converted the 5 Leapfrog grades and 5 USN/HC/HG star system to a 1 to 5 scale, 5 being the best rating. We defined discordance as any difference between the scores obtained in different databases and severe discordance as a larger than one scale difference (e.g., getting an A on Leapfrog and a 3 star on HC). We used contingency tables to evaluate the amount of discordance between different databases. We calculated the Spearman correlation factor for the correlation strength between variables. We used the SPSS IBM 28.0 software (Chicago, IL, USA) to perform all analyses.

## RESULTS

### Hospitals Characteristics

There were 3,871 hospitals in the American Hospital Directory database. Of those, 2,384 met our study's inclusion criteria. Hospitals were mainly in the South (940, 39.4%), followed by the Midwest (567,23.8%), West (484, 20.3%), and Northeast (393,16.5%). Appendix Table 5 lists the number of hospitals by state, and Appendix 2 lists the states attributed to each region. The average number of beds was 197 (± 237), the average number of yearly discharges was 7,754 (± 10,835), and the average number of patient days was 37,936 (± 64,212).

### HC and Leapfrog’s Overall Ratings

The Leapfrog Hospital Safety Grade distribution showed 688 (29%) with A, 652 (27.3%) with B, 885 (37.1%) with C, 153 (6.4%) with D, and 6 (0.3%) with F. As for the HC stars, 333 hospitals (14%) had five, 676 (28.4%) four, 695 (29.2%) three, 502 (21.4%) two, and 171 (7.2%) one. Table 1 shows the concordance of the HC star rating and Leapfrog Hospital Safety Grade. The ratings were discordant 70% of the time (difference of one or more) and severely discordant (difference of 2 or more) 25.1% of the time (598 hospitals). There was a very weak correlation between the Leapfrog and HC ratings, with a Spearman's correlation coefficient of 0.37[0.33–0.4], *P* < 0.001.

**Table 1:** Concordance of HC and Leapfrog Ratings (Number of Hospitals in Each Category)

HC	Five stars	Four stars	Three stars	Two stars	One star	Total
Leapfrog						
A	181	247	137	79	8	688
B	82	209	218	123	20	652
C	66	195	270	255	99	885
D	4	24	33	52	40	153
F	0	1	1	0	4	6
Total	333	676	695	509	171	2,394
Only USN Ranked hospitals						
A	77	64	41	12	1	195
B	20	49	39	11	1	120
C	13	31	46	37	10	137
D	0	4	3	6	4	17
Total	110	148	129	66	16	469

### USN-Ranked Hospitals and Leapfrog and HC Ratings

USN ranked 469 hospitals (19.7%) regionally or nationally. The Leapfrog rating of the USN-ranked hospitals showed 195 (41.6%) with an A, 120 (25.6%) with a B, 137 (29.2%) with a C, 17 (3.6%) with a D, and zero with an F. The HC rating of the same hospitals showed 110 (23.5%) with five stars, 148 (31.6%) with four stars, 129 (17.5%) with three stars, 66 (14%) with two stars, and 16 (3.4%) with one star. Only 77 hospitals (3.2%) got a USN ranking, a Leapfrog grade A, and five stars on HC. Within the USN-ranked hospital group, discordance between HC and Leapfrog was 62%, and severe discordance was 19.8%. Two hundred ten hospitals got a USN ranking, A or B on Leapfrog, and five or four stars on HC (Table 1).

### USN High-Performing Hospitals and Leapfrog and HC Ratings (Table 2)

**Table 2:** Concordance of USN High-Performing Hospitals with Leapfrog and HC Ratings (Number of Hospitals in Each Category)

		Number of USN High-performing specialties			Total	Number of USN High-performing Conditions or Procedures				
		0	1	≥ 2		0	1	2–3	4–5	> 5
Leapfrog	A	569	45	75	688	122	88	128	106	244
	B	573	31	48	652	177	101	115	93	166
	C	816	26	43	885	306	142	146	105	186
	D	142	4	7	153	71	24	22	13	23
	F	6	0	0	6	6	0	0	0	0
Hospital Compare	5	248	31	54	333	72	41	54	38	128
	4	582	35	58	676	178	103	110	91	194
	3	628	25	42	695	201	121	122	91	160
	2	483	11	15	509	168	62	92	75	112
	1	164	4	3	171	63	28	33	22	25

USN has two additional distinction categories: high-performing specialties and high-performing conditions or procedures. Two hundred seventy-nine hospitals were high-performing in one or more specialties, with an average of two specialties per hospital (± 1.7), and1,702 hospitals were high-performing in one or more conditions or procedures, with an average of 5.16 (± 4.2) per hospital.

We divided hospitals into three groups based on the number of high-performing specialties: 2,105 (88.3%) had none, 105 (4.4%) had one, and 173 (7.3%) had two or more. Similarly, we divided the hospitals based on the number of high-performing conditions or procedures: 682 (28.6%) had none, 355 (14.9%) had one, 411 (17.2%) had two or three, 317 (13.3%) had four or five, and 619 (26%) had more than five.

Hospitals with high-performing specialties had a very weak correlation with Leapfrog (0.13[0.8–0.17] and HC 0.19[0.15–0.23]. Similarly, there was a weak correlation for hospitals with high-performing conditions or procedures with Leapfrog 0.21[0.17–0.25] and HC 0.13[0.8–0.17] (all p < 0.001). For example, as shown in Table 2, which displays the hospital number by rating category, many Leapfrog Grade C or HC 3-star hospitals were deemed high-performing for specialties or procedures. Conversely, many Grade A Leapfrog or 5-star HC hospitals did not have a single USN high-performing specialty or procedure.

### Surgical Events

Leapfrog and HG recorded events of surgical objects left in patients' bodies in 198 (8.3%) and 444 (18.6%), respectively, but only 164 hospitals (6.9%) had events in both Leapfrog and HG.

### 30-Day Survival Rates and Orthopedic Procedures Complications

USN and HG recorded 30-day survival rates for many conditions. Table 3 shows the degree of discordance between databases. For 30-day survival rates, discordance ranged from 28.3% to 52.5%, and severe discordance from 20.9% to 40.8%. For the ratings of orthopedic procedures, discordance was higher and ranged from 48% (hip replacement) to 61.2% (hip fracture), and severe discordance ranged from 35.6% (hip replacement) to 49.3% (hip fracture).

**Table 3:** Discordance Between Survival Rates and Complication Rating in USN and HG

	Hospital numbers reporting	Discordance*	Severe discordance**
30-day survival rates			
Heart Attack	1,928	898 (46.6%)	603 (31.3%)
Aortic or Valve surgery	711	201(28.3%)	152(21.4%)
Bypass surgery	886	315 (35.6%)	185(20.9%)
Heart failure	2,357	1207 (51.3%)	897(38.4%)
Colon or colon cancer surgery	1,816	478 (26.3%)	364(20%)
Stroke	1998	1048 (52.5%)	808 (40.4%)
COPD	2,278	939(39.4%)	620(27.2%)
Pneumonia	2,350	1,257(53.5%)	959(40.8%)
Orthopedic procedures ratings			
Hip Fracture	2,198	1,346(61.2%)	1,084(49.3%)
Hip Replacement	1,601	769(48%)	570(35.6%)
Knee Replacement	1,701	853(50.1%)	714(42%)

^*^ Difference between the scores obtained in different databases

^**^ Larger than one scale difference between scores

## DISCUSSION

Our study examined the hospital rating results of four publicly reporting entities that seek to determine how hospitals perform. We found discordance —often substantial— that may confuse patients and consumers using the data to make informed healthcare decisions.

To explain the discordance, we shall examine the differences between the four rating organizations (Table 4).^11–13,15^ First, the organizations have distinct interests and focus on different aspects of quality and safety. Their for-profit or nonprofit status might also impact some of the results. Second, each rating system records different data and chooses its unique methodology. Leapfrog asks each hospital to complete a survey twice yearly and states it validates findings using specific methods. USN has a unique ranking of hospitals’ reputations as assessed by clinicians in the field. As for the measure types used in the ranking, all four organizations include outcome measures, but the specifics vary. For example, HG only has risk-adjusted mortality for conditions and procedural complications. Structural data is unique to Leapfrog (use of CPOE, barcoding, physician staffing) and USN (nursing staffing), whereas HC includes efficiency and timeliness of care.

**Table 4:** Characteristics of Hospital Rating Organizations

	HC	HG	Leapfrog	USN
Type of organizations	Nonprofit	For-profit	Nonprofit	For-profit
Financing consultative services/fees	Potential financial incentive for participating hospitals (reduced Medicare fee for service payment rate)	Hospitals pay a fee if using the rating for marketing purposes. Offers consulting services to hospitals	Hospitals pay a fee if using the rating for marketing purposes	Hospitals pay a fee if using the rating for marketing purposes
Measures and metric distribution	Process (Timely and Effective Care) 12% Outcomes – 88% (mortality 22%, safety of care 22%, readmissions 22%, patient experience 22% The distribution can change if some data is not submitted	Outcomes	Structural and process 50% Outcome 50%	Structure, process, and outcome measure distribution is not transparent
Inclusion Criteria	Majority of acute care facilities	All Medicare participating hospitals	~ 2,600 acute care hospitals	Meet one of four eligibility requirements
Result type	Quarterly rating, 1 to 5 stars	Actual performance compared to predicted performance using a risk-adjusted model results in a star rating Annual ranking of best 50, 100, 250 hospitals	A calculated weighted z-score translates to a biannual letter grade	Market research firm completes rankings annually

When comparing USN, HG, and Leapfrog, we found different results for specific metrics, such as surgical events or 30-day mortality. The self-reporting nature of Leapfrog may include inaccuracies. However, for the rating process to be transparent, the risk-adjusted methodologies and studied timelines should allow full replication and estimation of results and grades.

The findings of our study are consistent with previous work. Halasyamani compared the performance of USN and HC in acute myocardial infarction, congestive heart failure, and community-acquired pneumonia and found significant discordance between the ranking systems.^9^ Austin et al. analyzed four national rating systems (*USN, Leapfrog, H*G, and *Consumer reports®*) and found that only 10% of 844 hospitals rated by one system as high-performing were also rated similarly by another.^3^ The ranking analysis from five national rating systems (*Leapfrog ®, Vizient®, Truven®, Hospital Compare ®,* and *US News*®) found only a weak correlation between the ratings. One shortcoming of that study is that it only analyzed a small group of hospitals ranked at the top. Furthermore, the *Vizient* ® data is only available to subscribers.^8^

Unlike previous studies, ours included all adult hospitals, not only the highly ranked ones, making it, to our knowledge, the largest to date. We did not focus solely on the overall rating. Our analysis of specific variables, such as survival and complications, was unique.

Our results and the findings of previous work show that the time has come to reflect critically on hospital rankings and their meaning for the public. Patients might struggle to grasp the reasons behind the rating differences, such as the type of data reviewed or the weight placed on each metric. As seen in our results, there was severe discordance in the classifications of hospitals more than a quarter of the time. Research on how patients utilize these publicly reported databases shows that less than half of them considered online reviews important when choosing a physician—with even lower use of online reviews in patients under 65 years.^16^ This conflicting data that is publicly available online might cast an overall suspicion of the entire process. The difficulty of understanding data and the vagueness and complexity of the metrics are some of the issues that limit the usefulness of hospital quality ratings.^17,18^ Harmonizing the ratings to make them understandable and meaningful for the patient or hospital is needed.^18^

One concern is that ratings often rely on 12 to 24-month-old data. From a practical point of view, logistics and data calculation are significant barriers. But how valuable would it be for a patient to know that a hospital had an excellent safety record two years ago if its performance has since worsened?

The Agency for Healthcare Research and Quality (AHRQ) defines quality of care as safe, timely, equitable, effective, efficient, and patient-centered.^19^ However, only HC includes efficiency and effectiveness in reporting cost and throughput. Many of the measures included in the rankings are likely not meaningful to patients.^18^ Patients might not care whether a hospital performs too many CT scans if outcomes such as mortality or infections are desirable.^20^ Hence, ranking organizations have an opportunity for a more patient-centered distribution and weighing of their measures.^21^

Our study highlights the need for more research to understand the consumers’ preferences when utilizing hospital ratings for their healthcare choices. A single unified reporting system might seem ideal, but it is unrealistic, given the different rating organizations. Furthermore, consumers might be used to, if not enjoy, browsing multiple websites. Some might also refer to websites like Facebook or Yelp, where other patients might have left hospital reviews. As a result, consumers might need assistance navigating the complex, widespread, and often discordant data. Instead of a single rating system, a single website that pulls the different ratings into one location and attempts to provide a unifying interpretation of the chaos could prove much more helpful.

We have thus far discussed the impact of discrepant ratings on patients and customers. We must also highlight the impact of ratings on hospitals. Wallenburg et al.^22^ studied three teaching Dutch hospitals and showed that rankings, with their high volatility, are criticized for faulty design and inability to improve performance. Yet, hospital managers and professionals meet them with ambivalence due to their concern about reputation and competition. Rankings pushed hospitals to invest in forms, introducing different information technologies, training and disciplining clinical staff to collect and register indicator information, and standardizing care processes to enable data collection. The hospitals' criticisms are reminiscent of recent news of colleges ending their participation in USN rankings.^23^

Our study has several strengths, including the large number of hospitals analyzed and the variables included. It also has several limitations. First, we only limited our search to adult general hospitals and, given the limitations in publicly available data, could not review every hospital in the USA. Second, we had to include hospitals with Leapfrog ratings that did not share their findings. Third, we based our conclusions on the data we collected during the research. We may have gotten different results if we performed the search other times. Our search method, however, mimicked what a patient or consumer would do, i.e., look at various ratings of one hospital simultaneously. The rating organizations’ methodologies often change, and given the lack of transparency, we cannot predict how the change will affect the discrepancies. Therefore, we can make a case for frequent analysis of their results to keep the consumer informed.

## CONCLUSION

The ratings of four organizations were significantly discordant on quality metrics, overall safety rankings, 30-day survival, and orthopedic procedure complications scores. Differences in methodology, time periods, and analyzed patient populations can explain the discrepancies. Still, the discordances that hospitals have criticized may create significant confusion for patients and consumers. Future research should understand the consumers’ needs and attempt to help navigate the discordant data to prevent confusion.

## Appendix 1

Table 5

**Table 5:** Number of Hospitals by State

		Frequency	Percent
Valid	AK	6	.3
	AL	49	2.1
	AR	28	1.2
	AZ	43	1.8
	CA	230	9.6
	CO	38	1.6
	CT	25	1.0
	DC	5	.2
	DE	5	.2
	FL	145	6.1
	GA	71	3.0
	HI	11	.5
	IA	28	1.2
	ID	11	.5
	IL	97	4.1
	IN	66	2.8
	KS	31	1.3
	KY	49	2.1
	LA	40	1.7
	MA	48	2.0
	MD	38	1.6
	ME	15	.6
	MI	63	2.6
	MN	39	1.6
	MO	57	2.4
	MS	32	1.3
	MT	10	.4
	NC	75	3.1
	ND	6	.3
	NE	17	.7
	NH	13	.5
	NJ	59	2.5
	NM	16	.7
	NV	17	.7
	NY	116	4.9
	OH	97	4.1
	OK	40	1.7
	OR	31	1.3
	PA	103	4.3
	RI	8	.3
	SC	41	1.7
	SD	10	.4
	TN	63	2.6
	TX	173	7.3
	UT	24	1.0
	VA	67	2.8
	VT	6	.3
	WA	39	1.6
	WI	56	2.3
	WV	19	.8
	WY	8	.3
	Total	2384	100.0

## Appendix 2

### States Categorized by Regions

Northeast: Connecticut, Maine, Massachusetts, New Hampshire, New Jersey, New York, Pennsylvania, Rhode Island, Vermont.

South: Alabama, Arkansas, Delaware, Florida, Georgia, Kentucky, Louisianna, Maryland, Mississippi, North Carolina, Oklahoma, South Carolina, Tennessee, Texas, Virginia, West Virginia.

Midwest: Illinois, Indiana, Iowa, Kansas, Michigan, Minnesota, Missouri, Nebraska, North Dakota, Ohio, South Dakota, Wisconsin.

West: Alaska, Arizona, California, Colorado, Hawaii, Idaho, Oregon, Montana, New Mexico, Nevada, Utah, Washington, Wyoming.
